# Australian Podiatry Research in Paediatrics: A Bibliometric Analysis

**DOI:** 10.1002/jfa2.70143

**Published:** 2026-03-12

**Authors:** Helen A. Banwell, Peta Tehan, Matthew Carroll, Cylie M. Williams

**Affiliations:** ^1^ School of Allied Health and Human Performance Adelaide University Adelaide South Australia Australia; ^2^ School of Clinical Sciences, Faculty of Medicine, Nursing and Health Sciences Monash University Melbourne Victoria Australia; ^3^ Auckland University of Technology Auckland New Zealand; ^4^ School of Primary and Allied Health Monash University Frankston Victoria Australia

**Keywords:** allied health, Australia, child, foot, publications, research trends

## Abstract

**Background:**

The purpose of this study was to undertake a bibliographic analysis of foot and lower leg research relating to paediatric podiatry by Australian or affiliated Australian authors.

**Methods:**

The Scopus database search was conducted to identify all foot and lower limb research articles involving an Australian cohort of participants, published by Australian authors, or those affiliated with Australian institutions, pertaining to paediatric podiatry, in English from 1970 to 2024. We used bibliometric analysis through an open‐source tool based on the R language. We described citations, journals, authors and institutions; countries and publications were manually categorised according to research type, level of evidence and funding source.

**Results:**

The search strategy yielded 280 eligible articles, which received a total of 8331 citations and were published by 793 authors in 104 journals. The most frequent journal was *Gait & Posture* (35 articles; 12%), and the most published institution was the University of Sydney (170 affiliations). Most of the Australian paediatric articles published focused on detection, screening and diagnosis (*n* = 70, 25%) and only 33 articles (12%) provided Level I evidence. Seventy‐three paediatric articles (25%) received Category 1 funding; 154 articles (55%) reported no research funding.

**Conclusion:**

Paediatric podiatry research represents 17% of Australian foot and lower limb research. Despite the smaller population base, paediatric research attracts a high level of engagement, moderate citation rates and low funding rates when compared to adult population studies. Paediatric podiatry research is primarily produced via Level 3 evidence. This highlights the need for an increase in the robustness of research methodologies in paediatric podiatry research to strengthen the quality and applicability of evidence informing clinical care for children and adolescent populations.

## 1 Background

Many foot and leg disorders present in childhood. In Australia, just over 2% of general practitioner encounters were with children and their caregivers seeking consultation for painful foot and leg conditions such as injury, infection, and concerns with the skin or nail, apophysitis, and lower limb pain [1]. Other common presentations to health professionals include an atypical foot or leg shape (such as flat feet or in‐toeing) [1, 2] and walking and running concerns [1, 2]. Foot health in children and adolescents is often compromised by systemic concerns, with one in 5 presenting with chronic pain [3], one in 3 classified as hypermobile [4] and approximately one in every 30 with a measurable developmental coordination disorder [5, 6]. Of note, all of these concerns decrease school attendance [7], social participation and physical function [8, 9] but more so when the lower limb is involved [10]. Concurrently, they also increase childhood obesity risk and mental health concerns, including suicidal ideation, which, in Australia, continues to be a common cause of death in males under the age of 45 years [11, 12, 13].

Podiatry plays a crucial role in the assessment and management of foot and lower limb disorders in children. There is growing evidence that common podiatric interventions such as orthoses, insoles or footwear can alter biomechanical parameters in flexible flat feet [14], improve balance in motor control issues [15], decrease the incidence of growing pains [16] and can improve gait and function in children with cerebral palsy [17]. Research has also identified that foot orthoses have limited effectiveness in children's flat feet where pain or function concerns do not exist [18]. Despite the impact podiatry can have on children's feet and lower limbs, paediatric training has limited hours within the preregistration curriculum [19] and only half interest in sports or high‐risk industry‐led education after registration (163 of 1135 (14%) versus. 278 of 1135 (24%) and 270 of 1135 (24%) respectively) [20]. Further, as children's foot and lower limb concerns differ from adult concerns, much of the existing podiatric‐related evidence is limited for this population. Research funding is far more commonly attributed to childhood cancer, maternal health, or prevention and/or slowing of neuromuscular conditions that impact children disproportion to adults [21]. Indeed, little is known about paediatric‐based foot and lower limb research despite its potential to improve development, mental health and social satisfaction. Therefore, this study aimed to explore the landscape of Australian paediatric research through a bibliometric analysis. This analysis of paediatric focused research was conducted as part of a national podiatry research priorities’ initiative [22]. The broader initiative was focused on understanding the research landscape across all areas of podiatry. Within this targeted analysis, we aimed to identify the researchers, topics and sources of funding that support paediatric podiatry research in Australia.

## 2 Method

This research was a bibliometric analysis of peer‐reviewed articles published from January 1970 to December 2024. We used the data from the Scopus database (Elsevier, Amsterdam, the Netherlands). This decision was based on the coverage of the Scopus database [23, 24]. Our study is reported using the bibliometric analysis guidelines [25].

### 2.1 Search Strategy

The search strategy included four concepts: (i) children and adolescents; (ii) walking and balance; (iii) common paediatric lower limb conditions of the foot and the leg and (iv) feet and footwear. Full details of the search strategy are provided in Supporting Information S1.

### 2.2 Article Selection

Titles and abstracts of peer‐reviewed articles were downloaded from the Scopus database. These were exported into Covidence (Veritas Health Innovation, Melbourne, Australia), an online systematic review programme. All titles, abstracts and full text articles were independently screened by two of the five authors or research assistants (HB, HU, CMW, PT or MC). Disagreements were resolved by author discussion or by the third researcher from the team. We did not calculate inter‐rater reliability during screening due to the number of reviewers contributing to screening. Articles were included if the publication was original research or systematic reviews, with at least one researcher having an Australia education or healthcare affiliation; participants were located in Australia and were published in English. Additional criteria were placed on systematic reviews. This included that the first or last author was required to have an Australian affiliation, to reduce potential overestimation of “Australian‐affiliated” publications related to co‐authorship. We deemed eligible research to be clinically relevant if applicable to paediatric podiatry scope of practice. For example, where the population was children with cerebral palsy, we deemed it outside of scope of practice if the intervention was only botulinum Toxin A, whereas it was included if it described an intervention of, or comparison with, serial casting. Aligning with the research aim, we also focused on research designs directly translatable to clinical practice rather than preclinical research such as laboratory‐based studies exploring foundational concepts. We also excluded case studies, research letters, guidelines, editorials, consensus documents, commentaries and conference abstracts.

### 2.3 Data Extraction

All articles were imported into Biblioshiny (*bibliometrix* package version 2.2.1, University of Naples Federico II, Naples, Italy) [26]. We used this programme to extract the following information from each article: year of publication, journal name, 2023 Impact Factor (using Journal Citation Reports [Clarivate Analytics, Philadelphia, Pennsylvania, USA]), number of citations (as recorded in the Scopus database [Elsevier, Amsterdam, Netherlands]), author names and institutional and country affiliation.

### 2.4 Data Synthesis

Our data synthesis methods and details of the classification systems are described in overarching research [22]. We firstly categorised the research type using the United Kingdom Clinical Research Collaboration (UKCRC) Health Research Classification System [27]. Each study was then manually assigned a level of evidence using the National Health and Medical Research Council (NHMRC) criteria [28] We also documented the funding sources according to the Australian Government Higher Education Research Data Collection (HERDC) specifications [29].

## 3 Results

### 3.1 Article Characteristics

**FIGURE 1:**
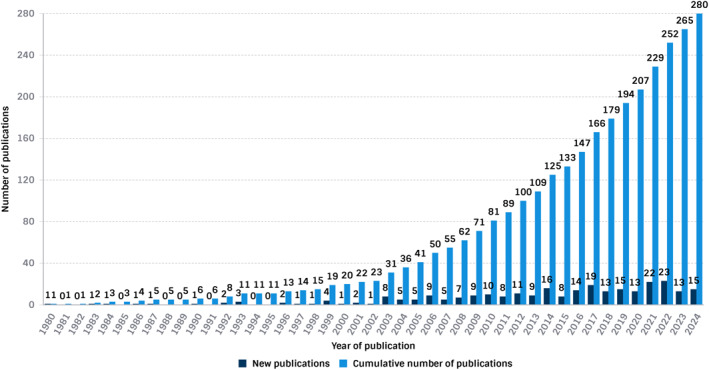
Number of articles (cumulative and per year).

The search strategy yielded 280 eligible articles (see Supporting Information S2) and the paediatric‐related article characteristics are summarised in Table 1. These included articles received a total of 8331 citations and were published by 793 authors. The top 10 most frequent journals were *Gait & Posture* (*n* = 33), *Journal of Foot and Ankle Research* (*n* = 17), *Developmental Medicine & Child Neurology* (*n* = 13), *Journal of Science and Medicine in Sport* (*n* = 9), *Journal of Pediatric Orthopaedics* (*n* = 8), *British Journal of Sports Medicine* (*n* = 7), *Journal of the American Podiatry Medical Association* (*n* = 7), *Clinical Biomechanics* (*n* = 6), *Foot* (*n* = 6), and the *Journal of Paediatrics and Child Health* (*n* = 6) (Table 2). Figure 1 shows the number of paediatric articles (cumulative and per year) from 1970 to 2024. The first publication relating to paediatric podiatry was not observed until 1980, with consistently increasing output since 2002.

**TABLE 1:** Publication characteristics.

	Paediatrics
Years	1980–2024
Total number of articles	280
Original articles	254
Systematic reviews	26
Mean years from publication	11.10
Mean citations per year per article	29.6
Citations	8331
Total authors	793
Mean co‐authors per article	5.1
International co‐authorships (%)	26.8
Single‐authored publications	5

**TABLE 2:** Top 10 most frequent journals.

Sources	*n* (%)	Quartile	IF (2023)
Gait & Posture	33 (12)	Q1	2.4
Journal of Foot & Ankle Research	17 (6)	Q2	2.9
Developmental Medicine & Child Neurology	13 (5)	Q1	4.3
Journal of Science & Medicine in Sport	9 (3)	Q1	3.4
Journal of Paediatric Orthopaedics	8 (3)	Q3	1.5
British Journal of Sports Medicine	7 (2)	Q1	16.3
Journal of the American Podiatry Medical Association	7 (2)	—	—
Clinical Biomechanics	6 (2)	Q3	1.4
Foot	6 (2)	—	—
Journal of Paediatric & Child Health	6 (2)	Q3	1.4

Abbreviation: IF, impact factor.

### 3.2 Authors, Institutions and Countries

The top 10 most frequent authors of the articles are presented in Table 3, with the five most published authors identified as Williams, CM., Burns, J., Steele, JR., Gray, K., Graham, HK. and Pacey, V. The most frequently represented affiliations are listed in Table 4, with the top five being the University of Sydney (*n* = 170), University of South Australia (*n* = 77), Monash University (*n* = 76), University of Melbourne (*n* = 60) and Curtin University (*n* = 45).

**TABLE 3:** Top 10 most frequent authors.

Author	Articles *n*, (%)
Williams CM	39 (14)
Burns J	37 (13)
Steele JR	14 (5)
Gray K	12 (4)
Rose KJ	12 (4)
Graham HK	11 (4)
Pacey V	11 (4)
Banwell HA	10 (4)
Evans AM	10 (4)
Wojciechowski E	10 (4)

**TABLE 4:** Top 10 most frequent affiliations.

Rank	Institution	Number of publications
1	University of Sydney	170
2	University of South Australia	77
3	Monash University	76
4	University of Melbourne	60
5	Curtin University	45
6	La Trobe University	39
7	University of Queensland	39
8	University of Wollongong	33
9	Children's Hospital at Westmead	32
10	Murdoch Children's Research Institute	30

### 3.3 Citations

Table 5 presents the top 10 most highly cited articles according to total citations and citations per year. Articles with the five highest total citations included a gait profile and movement analysis assessment (score) [30], a systematic review on the impact of strength training for young people with cerebral palsy [31], a pilot repeated‐measure study identifying impact of functional strength training in cerebral palsy [32], investigations of psychometric properties of scales related to measuring muscle and joint performance of children with cerebral palsy [33] and a randomised control trial comparing botulinum Toxin A and serial casting for calf tightness in children with cerebral palsy [34].

**TABLE 5:** Top 10 cited articles (total citations and average citations per article per year).

Authors (year)	Publication title	Journal	Citations (*n*)	Average citations per year
Baker (2009)	The gait profile score and movement analysis profile	Gait & Posture	454	28.38
Dodd (2003)	A randomised clinical trial of strength training in young people with cerebral palsy	Developmental Medicine and Child Neurology	233	10.4
Blundell (2003)	Functional strength training in cerebral palsy: A pilot study of a group circuit training class for children aged 4–8 years	Clinical Rehabilitation	174	7.91
Fosang (2003)	Measures of muscle and joint performance in the lower limb of children with cerebral palsy	Developmental Medicine and Child Neurology	173	1.60
Flett (1999)	Botulinum toxin a versus fixed cast stretching for dynamic calf tightness in cerebral palsy	Journal of Paediatrics and Child Health	171	1.86
Mickle (2006)	The feet of overweight and obese young children: Are they flat or fat?	Obesity	160	2.7
Shorter (2013)	Cochrane review: Screening programmes for developmental dysplasia of the hip in newborn infants	Evidence‐Based Child Health	156	3.88
Evans (2003)	Reliability of the foot posture index and traditional measures of foot position	Journal of the American Podiatric Medical Association	136	1.26
Lythgo (2009)	Basic gait and symmetry measures for primary school‐aged children and young adults whilst walking barefoot and with shoes	Gait & Posture	135	1.26
Mckay (2017)	Spatiotemporal and plantar pressure patterns of 1000 healthy individuals aged 3–101 years	Gait & Posture	131	3.65

### 3.4 Research Types and Level of Evidence

Using the UKCRC criteria, 107 articles (38%) were focused on detection, screening and diagnosis, 79 (28%) on evaluation of treatments and therapeutic interventions, 65 (23% on aetiology and 13 (5%) on underpinning research. There was only one (0.3%) study that reviewed prevention of disease and conditions and promotion of well‐being and one (0.3%) that investigated health and social care services. When the NHMRC levels of evidence was applied, there were 33 articles (12%) providing Level I evidence, 19 (7%) providing Level II evidence, 181 (65%) providing Level III evidence and 47 (17%) providing Level IV evidence.

### 3.5 Sources of Funding

One hundred and fifty‐four articles (55%) did not report research funding. The most common source of funding reported in the remaining articles was from Category 1 funding (*n* = 73; 26%), 41 (15%) from Category 3 funding and 12 (4%) from Category 2 funding, whereas no funding was reported from Category 4 (*n* = 0; 0%).

## 4 Discussion

This research identified the most active paediatric podiatry related researchers and institutions publishing 280 articles between 1980 and 2024 noting several key Australian authors and institutions. This explicitly aligns with the research aim. Our analysis revealed that paediatric podiatry research represents a good proportion of Australian podiatry research and focuses predominantly on detection, screening, and diagnosis, and a reasonable amount of research (25%) is supported by Category 1 (competitive) grant funding; however, 55% of the research is unfunded. These findings highlight both the high level of researcher engagement with the topic, and the predominantly underfunded nature of Australian paediatric podiatry research.

Interestingly, the total yield of paediatric related articles is the third highest of the topic areas represented in our larger bibliographic analysis (only behind diabetes and musculoskeletal‐related research) and represents 17% of research activity relevant to podiatry in Australia [22]. This is heartening as it suggests the foot and lower limb health of children and young people is given appropriate regard within the podiatry research space. This also mirrors clinicians' interest in topics, with the topic of paediatric education also claiming third preference for continuing professional development opportunities [20]. Paediatrics was the second highest cited topic (8331 citations across 280 articles), only behind musculoskeletal (with 13,697 citations across 308 articles); however, when considering citations per article, paediatrics (at 29.6 citations per year per article), sit behind neurovascular, musculoskeletal and gerontology at 28.8, 44.5 and 62 citations, respectively. This suggests that, on average, paediatric podiatry‐related articles receive only moderate interest from researchers.

Paediatric podiatry–related research was disseminated across a broad array of journals, predominantly within the gait and biomechanics, sports, and neurology fields, with only a proportion being published in paediatric‐focused journals. The higher impact publications appeared in journals focused on biomechanics, neurological, rehabilitation and obesity topics. Although this dissemination strategy may encourage an extended audience, there may also be a disconnect between those seeking paediatric podiatry–related evidence and journal readership, potentially impeding the translation of research evidence into clinical practice amongst those working more closely in the paediatric field. It may be prudent for researchers to consider the audience further when deciding on dissemination strategies. Of note, five articles were published within the Cochrane review library [18, 35, 36, 37, 38], ensuring that the best evidence, at the time of publication, related to management of talipes equinovarus, toe walking, using foot orthoses for children with flat feet, and ankle range of motion in neuromuscular disease, is freely available to all with interest.

Funding of paediatric podiatry–related research was limited and inconsistently reported. Fifty‐five percent of articles identified that no funding was gained to support the development of their research. Although this is consistent with other podiatry‐related bibliometric analysis [39, 40], it is plausible that a lack of funding contributes to the lower levels of evidence generated within this topic. Using NHMRC levels of evidence, only 33 articles (12%) provided Level I evidence (systematic review of all relevant randomised controlled trials) and only 19 (7%) provided Level II evidence (evidence from at least one properly designed randomised controlled trial) with nearly one‐third (*n* = 181, 65%) reporting on Level III evidence (e.g., nonrandomised controlled studies such as cohort or case‐controlled studies). Randomised trials offer the most robust outcomes but can be costly, time‐consuming and difficult to administer without adequate funding. Improving confidence in the body of evidence that guides clinical practice related to paediatric foot and lower limb health will require appropriate financial investment.

These outcomes need to be considered against several methodological limitations. Firstly, our criteria excluded case studies, conference abstracts, guidelines, consensus statements, letters, editorials and commentaries and therefore did not capture any relevant paediatric‐related research within these documents accordingly. Secondly, categorising articles into a single research classification also limits our ability to report the full scope of paediatric‐related research focus. Thirdly, funding information was inconsistently reported, and frequently failed to classify research related costs separately to researcher costs (e.g., salary and oncosts), limiting confidence that we are reporting the true financial investment. Finally, we acknowledge that citation metrics, despite being a useful indication of researcher interest, do not necessarily identify the quality of the research or the impact it has on clinical practice and outcomes for foot or lower limb concerns related to paediatrics.

In summary, our bibliometric analysis highlights paediatrics podiatry–related research attracts a high level of engagement within Australian‐led, focused or affiliated foot and lower limb research. This analysis also highlights its importance in the podiatry profession. Although articles in this field have accrued a high total citation count, individual articles are only moderately cited on an annual basis. This is only one translation metric, but it suggests limits to translation and visibility of paediatric podiatry research. Our analysis also identified constrained funding and potential misalignment between journal selection and the intended readership. Limited funding and publication in journals not routinely accessed by podiatrists may further restrict the translation of evidence into practice. Targeted investment and podiatrist‐focused dissemination strategies may continue to strengthen evidence‐based paediatric podiatry practice. Further, the development of appropriately focused Australian paediatric podiatry research priorities would support opportunities to champion research across the lifespan, in addition to improving evidence translation within podiatry education and practice.

## Author Contributions

**Helen A. Banwell:** conceptualization, funding acquisition, data curation, writing – original draft, writing – reviewing and editing. **Peta Tehan:** conceptualization, project administration, methodology, formal analysis, writing – review and editing. **Matthew Carroll:** conceptualization, methodology, formal analysis, writing – review and editing. **Cylie M. Williams:** conceptualization, data curation, writing – original draft, writing – reviewing and editing.

## Funding

The study was supported by Australian Podiatry Education and Research Foundation.

## Conflicts of Interest

Cylie Williams is an associate editor for the Journal of Foot and Ankle Research but was not involved with the handling of this paper. There are no other conflicts to declare.

## Supporting information


Supporting Information S1

Supporting Information S2

## Data Availability

Data are available from the corresponding author on reasonable request.
