# Supplementary material for: Australian Podiatry Research in Paediatrics: A Bibliometric Analysis
Source: J Foot Ankle Res. 2026 Mar 12;19(1):e70143. doi: 10.1002/jfa2.70143 (PMC13097461; doi:10.1002/jfa2.70143)
Supplement: Supplementary file 1 — Supporting Information S1 [file JFA2-19-e70143-s002.docx]

| **Paeds** | Search Strategy Scopus |
| --- | --- |
| 1 | paed* OR child* OR teen* OR adoles* OR infant OR toddler OR pre-school |
| 2 | pain OR footwear OR shoe OR "foot orth*" OR gait OR walk* OR run* OR balance OR assess* OR screening OR osteochondro* OR sever* OR perthe* OR "osgood" OR apophysit* OR "Growing pain*" OR "toe walk*" OR "intoe*" OR toe OR "Flat foot" OR "flat feet" OR "pes planus" OR "Pes planovalgus" OR "neuro*" OR "cerebral palsy" OR "charcot marie tooth" OR "Developmental coordination disorder*" OR "Motor coordination" OR arthritis OR hypermob* OR "Club foot" OR "Talipes" OR "Metatarsus add*" OR autism OR idio* |
| 3 | toe OR foot OR feet OR leg OR ankle OR knee OR hip OR pod* |
|  | 1 AND 2 AND 3 |
|  |  |
| **Search Restrictions** | |
| Year | 1970-2023 |
| Language | English |
| Source | Article |
| Author Affiliation | Australian |
